# Effects of 5‐year experimental warming in the Alpine belt on soil Archaea: Multi‐omics approaches and prospects

**DOI:** 10.1111/1758-2229.13152

**Published:** 2023-03-30

**Authors:** Federica D'Alò, Laura Zucconi, Silvano Onofri, Fabiana Canini, Nicoletta Cannone, Francesco Malfasi, Daniel Kumazawa Morais, Robert Starke

**Affiliations:** ^1^ Department of Ecological and Biological Sciences University of Tuscia, Largo dell'Università Viterbo Italy; ^2^ Institute of Polar Sciences National Research Council of Italy (CNR‐ISP) Messina Italy; ^3^ Department of Science and High Technology Insubria University Como CO Italy; ^4^ Biological Institute of São Paulo – Vila Mariana São Paulo Brazil; ^5^ Norwegian College of Fishery Science UiT the Arctic University of Norway Tromsø Norway; ^6^ Institute of Microbiology of the Czech Academy of Sciences Praha Czech Republic

## Abstract

We currently lack a predictive understanding of how soil archaeal communities may respond to climate change, particularly in Alpine areas where warming is far exceeding the global average. Here, we characterized the abundance, structure, and function of total (by metagenomics) and active soil archaea (by metatranscriptomics) after 5‐year experimental field warming (+1°C) in Italian Alpine grasslands and snowbeds. Our multi‐omics approach unveiled an increasing abundance of Archaea during warming in snowbeds, which was negatively correlated with the abundance of fungi (by qPCR) and micronutrients (Ca and Mg), but positively correlated with soil water content. In the snowbeds transcripts, warming resulted in the enrichment of abundances of transcription and nucleotide biosynthesis. Our study provides novel insights into possible changes in soil Archaea composition and function in the climate change scenario.

## INTRODUCTION

Over the 21st century, temperature changes are affecting ecosystems, communities, and species all over the world, with severe consequences for biodiversity and related ecosystem processes. Particularly Alpine areas, characterized by high environmental harshness, such as low temperature, acidic pH, and limited water content (Donhauser & Frey, 2018), are experiencing warming well above the global average and are expected to be more sensitive to climate change (Diaz et al., 2003; Donhauser & Frey, 2018; Thompson, 2000). Consequently, rising temperatures may result in changes of soil microbial communities composition and functioning related to biogeochemical processes such as carbon (C) and nutrients cycling (D'Alò et al., 2022; Yu et al., 2021; Yuan et al., 2021). These patterns cannot be investigated solely with ecosystems observation studies; indeed, multiple environmental factors may interact with each other, resulting in complex direct and indirect effects on all pools and their fluxes in all ecosystems (Cavaleri et al., 2015). In this light, manipulative studies can distinguish between correlation and causation, which is why field‐based climate warming manipulation experiments are frequently used (Cavaleri et al., 2015; Klein et al., 2005; Timonen & Bomberg, 2009). Such field experiments can isolate the effects of individual treatments to test mechanistic hypotheses, revealing important insights about ecosystem responses to extreme or abrupt climatic events (Klein et al., 2005; Timonen & Bomberg, 2009).

Noteworthy, most of the studies on the responses of microbial communities to experimental warming focus on bacteria and fungi, while knowledge on the abundance, diversity, and function of soil archaea, especially in high‐altitude environments is still scarce (Hofmann et al., 2016). However, Archaea are omnipresent in terrestrial environments (DeLong & Pace, 2001; Timonen & Bomberg, 2009), with key roles in the global carbon and nitrogen cycles (Leininger et al., 2006). Metabarcoding screening targeting the 16S rRNA gene region showed a relative archaeal abundance of up to 10% of soil prokaryotes (Bates et al., 2011; DeLong & Pace, 2001) peaking in extreme habitats, such as high acidic and high altitude ones (Cavicchioli et al., 2000; Korzhenkov et al., 2019). Recently, it was reported that the relative abundance of archaeal proteins ranged between 0.6% and 6.8% of total soil proteins, with a significant positive correlation with aridity index, which was in turn correlated with a loss of bacterial and fungal diversity (Starke et al., 2021). Methyl‐coenzyme M reductase (*MCRA*), tyrosine–tRNA ligase (*SYY*), and DNA protection during starvation (*DPS*) were identified among the most common functions across forest, grassland, and shrubland soils (Starke et al., 2021). *MCRA* is central to methanogenic pathways (Lyu et al., 2018) and is encoded by strictly anaerobic archaea converting a restricted number of substrates to methane (Hedderich & Whitman, 2006) and thereby potentially controlling methane production across climate (Starke et al., 2021). Not found in the proteins by Starke et al. (2021) but ubiquitous and abundant in the environment is the ammonia monooxygenase (amo) encoded by ammonia‐oxidizing archaea (AOA) (Könneke et al., 2005; Treusch et al., 2005; Venter et al., 2004), which play a key role in nitrification (Stahl & de la Torre, 2012), carbon fixation (Hansman et al., 2009), and methane production in oceans (Metcalf et al., 2012). On a higher level of functionality, harsh soils are characterized by a large number of markers involved in the maintenance of cellular and high growth potential (e.g. higher metabolic quotients and genes related to central carbon metabolism, biosynthesis, DNA replication, transcription and translation) in addition to the traits involved in stress tolerance (Lyu et al., 2018).

We used metagenomics and metatranscriptomics to investigate the responses of the soil archaeal community to a 5‐year in situ experimental warming (using open top chambers [OTCs]) in Alpine grassland and snowbed (D'Alò et al., 2021). We hypothesized (i) a higher abundance of archaea in snowbeds than in grasslands due to their ability to adapt to the lower temperatures and harsher environmental conditions characterizing the snowbed belt; (ii) an increased abundance of archaea upon warming compared to controls due to their ability to grow in more arid conditions; and (iii) functional differences between vegetation belts and upon warming.

The selected area was located around the Stelvio Pass in the Stelvio National Park, Italian central Alps (46°31′ N, 10°25′ E), due to evidence of recent climate change which included vegetational changes since 1953, such as shrub upwards migration and encroachment at the expense of Alpine grasslands and snowbeds (Cannone et al., 2007). The selected Alpine belt (2604–2624 m a.s.l.) comprised two different plant communities: a climax Alpine grassland (*Caricetum curvulae*) and an Alpine snowbed (*Salicetum herbaceae*). Both plant communities are highly vulnerable to the impacts of climate change and are at high risk of regression due to the ingression of species from neighbouring communities (Cannone et al., 2007; Cannone & Pignatti, 2014; Malfasi & Cannone, 2021). To study the effect of short‐term warming, a manipulative experiment was conducted in situ (D'Alò et al., 2021, 2022; Sannino et al., 2022). In 2014, small hexagonal OTCs (2.08 m in diameter) were installed in both Alpine grassland and Alpine snowbed. OTCs passively increased the ambient summer temperature of 1°C (Malfasi & Cannone, 2021) to reach values predicted by the best future warming scenario (RCP2.6) by 2100 (IPCC, 2018). The paired plots outside the OTCs were used as controls, representing current conditions. Five soil samples were collected for each condition on the same day in July 2019 during the peak of the growing season. After removal of plant debris and roots, at least three soil scoops were passed through a 5‐mm sterile mesh and mixed to obtain a composite sample, which was collected in Falcon tube. Data for edaphic and environmental variables, bacterial and fungal abundance using qPCR, and raw metagenomic and metatranscriptomic data were obtained from a previously published paper focusing on the abundance, diversity, and function of bacteria and fungi (D'Alò et al., 2022). Briefly, nucleic acid samples for the metagenomics (MG) and metatranscriptomics (MT) were pooled in equimolar volumes and sequenced on an Illumina NovaSeq6000 SP (DS‐150) with a 2 × 150 bp configuration. MG assembly and annotation were performed as previously described (Žifčáková et al., 2017). Combined assembly was performed using MEGAHIT 1.1.3 (Li et al., 2015). Gene calling was performed using MG‐RAST (Meyer et al., 2008). Taxonomic identification was performed both using MG‐RAST identification based on Integrated Microbial Genomes (IMG) dataset as a reference and by BLAST against all published fungal genomes available in MycoCosm fungal genomics portal on January 2020 (Grigoriev et al., 2014). Of these two, that taxonomic identification with a higher bitscore was used as the best hit with minimal threshold of bitscore 54. The functions of the predicted genes were annotated with the hmmsearch function in HMMER 3.2.1 (Eddy, 2011) using the FOAM database as a source of HMMs for relevant genes (Prestat et al., 2014). To obtain the number of genes per sample, reads were mapped to the assembled contigs using the Bowtie 2 program (Langmead & Salzberg, 2012). Using the Kyoto Encyclopedia of Genes and Genomes (KEGG) Orthology database (Kanehisa & Goto, 2000), the dominant pathways were annotated at KEGG level 2. All analyses were performed in R (R Core Team, 2020). We extracted the archaeal genes and transcripts from these data as it is commonly done to describe prokaryotic communities (Clark et al., 2021; Kakuk et al., 2021; Souza et al., 2016). More information is available in the study by D'Alò et al. (2022) and the supplementary material S1.

## RESULTS AND DISCUSSION

**FIGURE 1:**
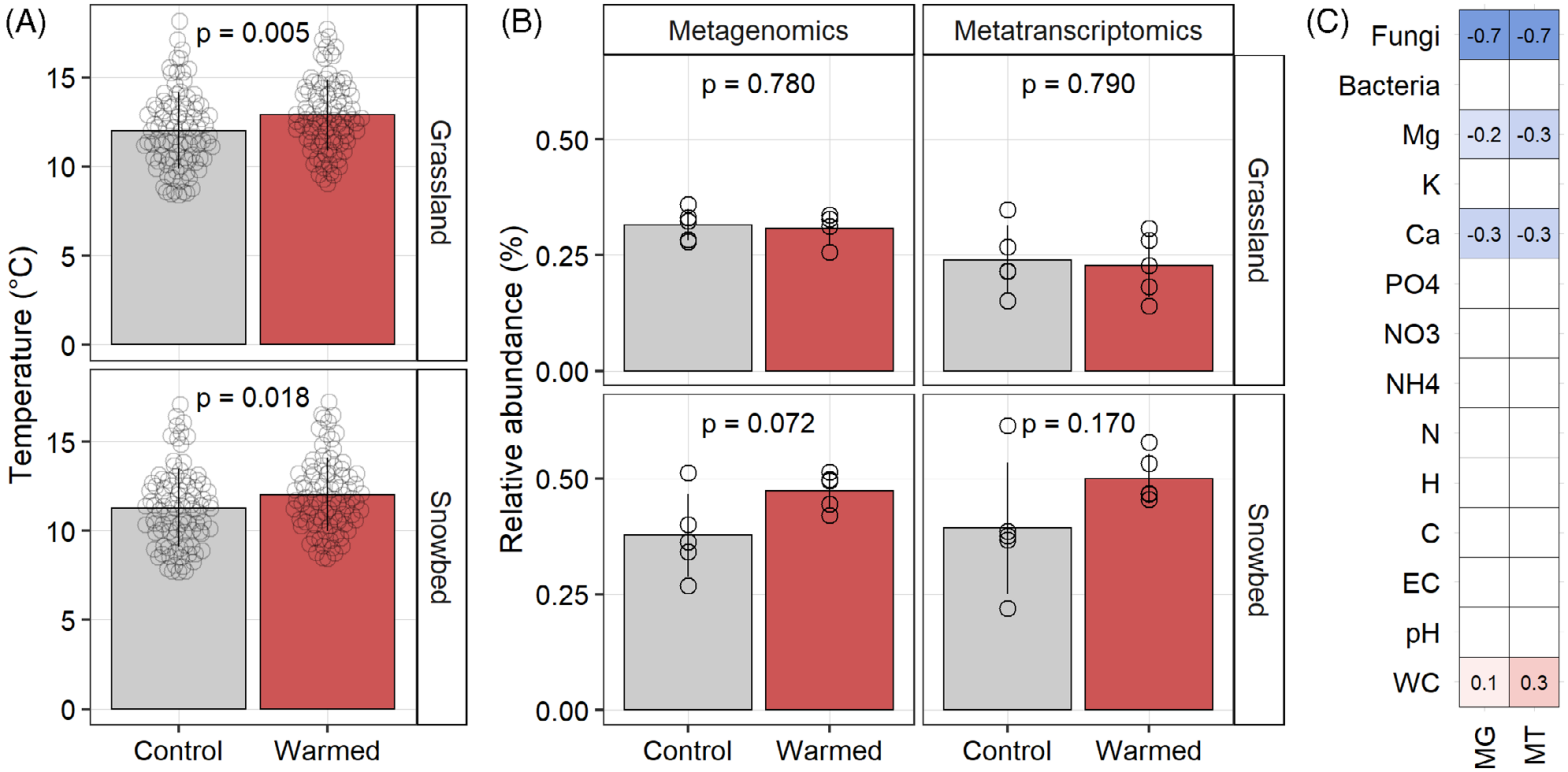
Temperature during the growing season in grasslands and snowbeds in control plots and during warming (A), and the relative abundance of archaeal genes and transcripts (B). *P* values were estimated as paired *t*‐tests. Significant (*p* < 0.05) spearman correlations of edaphic and environmental parameters as well as the abundance of bacteria and fungi based on qPCR against the relative abundance of archaea in genes (MG) and transcripts (MT) (C).

Experimental warming using OTCs successfully increased the ground surface temperatures of 1°C over 5 years (Figure 1A). Particularly, temperature increased from 12.0 ± 2.1°C in control plots to 12.9 ± 2.0°C under warming in grasslands and from 11.3 ± 2.2°C in control plots to 12.0 ± 2.1°C in snowbeds, both with statistical evidence. The number of genes and transcripts assigned to Archaea in grasslands was not significantly different between control and warmed plots, whereas a weak statistical increase in archaeal abundance was observed in warmed snowbed plots, in both MG and MT (Figure 1B). Control grassland samples showed a lower abundance of archaea genes (*p* = 0.2049) and transcripts (*p* = 0.0753) than control snowbeds, and warming increased the magnitude of this trend in both genes (*p* = 0.0004) and transcripts (*p* = 0.0002) in both vegetations (data not shown). Among the physicochemical parameters (Table S1), only water content (WC) showed a slight significant positive Spearman correlation with the abundance of archaeal genes and transcripts, while calcium and magnesium, among the micronutrients, and fungal abundance, showed a significant negative Spearman correlation (Figure 1C, Figure S1).

**FIGURE 2:**
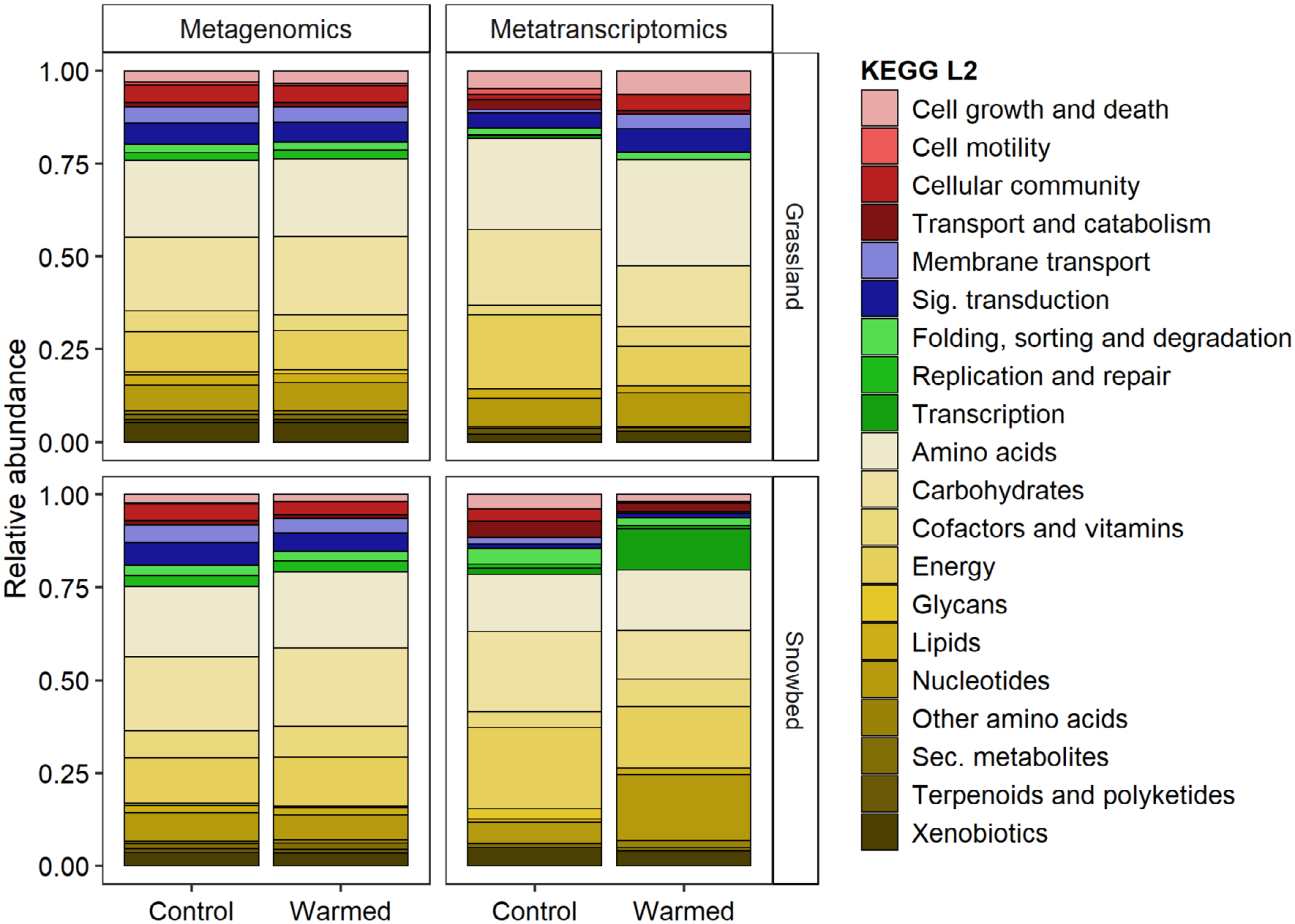
Relative abundance of KEGG level 2 (L2) functions assigned to archaea in grasslands and snowbeds in control plots and during warming. Cellular processes are shown in red, environmental information in blue, genetic information in green, and metabolism in yellow.

The functional profile of soil archaea showed the dominance of metabolic functions, as indicated by the yellow scale in Figure 2, regardless of sequencing method, vegetation belt, or treatment. Lower abundances were found for functions related to cellular processes, environmental information, and genetic information. However, only the genes in grasslands showed an almost identical functional composition in both control and warmed plots. Genes in samples subjected to warming in snowbeds comprised a higher number of functions related to metabolism, particularly of carbohydrates. Transcripts in grasslands showed a higher contribution of cellular processes and environmental information at the expense of genetic information and metabolism under warming compared to control plots. Otherwise, transcripts in warmed snowbeds plots showed a higher contribution of functions related to nucleotides and transcription associated to a reduction of cellular processes and environmental information, compared to the control plots. In line with our first hypothesis, we found a higher relative abundance of archaeal genes and transcripts in snowbeds than in grasslands. Indeed, snowbeds are characterized by harsher conditions favouring archaeal presence and activity, due to long‐lasting snow cover, longer periods of low soil temperature, and a shorter growing season (D'Alò et al., 2021, 2022).

Archaeal sequences abundance further increased with warming only in snowbeds. Aside from the increased soil temperature for both vegetations inside the OTCs, no differences in water content or other physicochemical parameters were observed between warmed and control plots. Therefore, unlike our second hypothesis, the expected increase of archaea in warmer plots does not appear to be a result of increased aridity.

The total archaeal relative abundances in our samples ranged from 0.26% to 0.51% in MG and 0.14% to 0.61% in MT. These values are much lower than those recorded in prokaryotic communities around the world using 16S rDNA gene amplicon sequencing (Bates et al., 2011; DeLong & Pace, 2001). However, in our study, both MG and MT also included sequences from non‐prokaryotic organisms and not just bacteria as in mentioned studies, which might explain the much lower relative contribution of archaea.

Archaea resulted to be negatively correlated with fungal biomass and micronutrients, implying that these organisms are more likely to be found in areas with low organic C, nutrient availability, and organic matter turnover (Burke et al., 2012). This appears to be consistent with previous findings in which an increase of fungi was recorded moving from Alpine to sub‐Alpine belts, as a result of more favourable environmental conditions (D'Alò et al., 2022).

The positive correlation of the whole pool of archaeal genes and transcripts with water content, on the other hand, seems to contradict the previously observed positive correlation between archaeal proteins and aridity in several ecosystems (Starke et al., 2021). In any case, a direct comparison is questionable because the aridity index is defined as the ratio of mean annual precipitation to mean annual evapotranspiration rather than water content. Water is essential to all life forms, however, linear regression showed only weak coefficients of determination for both genes (*R* ^2^ = 0.01) and transcripts (*R* ^2^ = 0.11), which is why manipulation experiments using different water contents would be necessary to test the validity of this correlation.

A dominance of Halobacteriota, Methanobacteriota, and Thermoproteota (Figure S2) was recorded. These taxa did not match the previously reported dominance of Nitrososphaerales (Thermoproteota), and Thermoplasmata (Thermoplasmatota) across different terrestrial ecosystems (Bates et al., 2011; Isoda et al., 2017; Neilson et al., 2017; Starke et al., 2021). In this study, not only did the annotation produce fewer archaeal sequences than expected based on a previous research, but the quality of the annotation appeared to be lacking, in particular for non‐cultivable taxa. Indeed, the community profile mirrored the profile from published genomes at JGI used for the annotation of the sequences, which is why we decided to remove community composition from this study. This seems to be a common issue of meta‐omic studies. Similarly, in a previous metaproteomic analysis of archaea, only the functional part of the metaproteomic data was published (Starke et al., 2021), being the database (https://www.uniprot.org) to which protein sequences are uploaded biased by the access to culturable strains. For archaeal taxonomy, to the current state, we suggest to use 16S rRNA gene amplicon sequencing instead.

Despite the lack of reliability in the taxonomic results, the general functionality should be equally well described in archaea as the general processes that we described are essential in any living organism. In line with our third hypothesis, we identified functional differences between vegetation belts and upon warming. A higher abundance of transcripts related to transcription and nucleotide biosynthesis during warming in snowbeds were observed, both considered related to strategies for maintaining high cellular growth potential under stress conditions (Diaz et al., 2003; Donhauser & Frey, 2018; Thompson, 2000). This difference was not visible in grassland transcripts, implying a greater impact of warming on archaea in more hostile environments (Diaz et al., 2003; Donhauser & Frey, 2018; Thompson, 2000). Despite the above results, no differences in the genes of both grasslands and snowbeds were observed, highlighting a difference between potential and real activity within the microbiome under different conditions. Apparently, only the active archaeal community changed its metabolic activity upon warming, which was consistent with the differential sensitivity of the soil microbiome in response to drought and forest management (D'Alò et al., 2022). Comparing between studies requires the use of similar functional classifications. In this study, we were only able to use KEGG for functional annotation, while in the work by Starke et al. (2021), the soil archaeal metaproteome was annotated by COG, making their comparison impossible.

Going deeper, investigating methane and nitrogen metabolisms or specific functions such as *MCRA* was impossible, due to the low number of archaeal sequences obtained. Certainly, these findings revealed the bottlenecks of investigating low abundant organisms in the soil food web, which could improve in the future when more archaeal genomes are sequenced.

To conclude, we investigated the impact of short‐term experimental warming by +1°C on the abundance, structure, and function of archaeal genes and transcripts in grasslands and snowbeds of the Italian Alpine belt. We found an increasing number of archaeal genes and transcripts in snowbeds but not in grasslands; moreover, an increased abundance was recorded upon warming in snowbeds, highlighting that warming is able to impact the soil microbiome, in particular archaeal community, starting from the more vulnerable, harsher, colder altitude site. A functional shift in the active but not the total snowbed community was unveiled during warming in line with the maintenance of high growth potential. However, due to the scarcity of archaea sequences, it was impossible to investigate specific functions, preventing us from going deeper into the ecological consequences of global ecosystem changes.

## AUTHOR CONTRIBUTIONS

**Federica D'Alò:** Conceptualization (equal); investigation (equal); writing – original draft (equal); writing – review and editing (equal). **Laura Zucconi:** Supervision (equal); writing – original draft (equal); writing – review and editing (equal). **Silvano Onofri:** Funding acquisition (equal); project administration (equal); writing – review and editing (equal). **Fabiana Canini:** Resources (equal); writing – review and editing (equal). **Nicoletta Cannone:** Methodology (equal); resources (equal); writing – review and editing (equal). **Francesco Malfasi:** Resources (equal); writing – review and editing (equal). **Daniel Kumazawa Morais:** Data curation (equal); writing – review and editing (equal). **Robert Starke:** Conceptualization (equal); formal analysis (equal); writing – original draft (equal); writing – review and editing (equal).

## CONFLICT OF INTEREST STATEMENT

The authors declare no conflict of interest.

## Supporting information


**Data S1:** Supporting Information

## Data Availability

The datasets MG and MT have been deposited on MG‐RAST under accession numbers mgm4901504.3 and mgm4901505.3, respectively.

## References

[emi413152-bib-0001] Bates, S.T. , Berg‐Lyons, D. , Caporaso, J.G. , Walters, W.A. , Knight, R. & Fierer, N. (2011) Examining the global distribution of dominant archaeal populations in soil. ISME Journal, 5, 908–917. 21085198 10.1038/ismej.2010.171 PMC3105767

[emi413152-bib-0002] Burke, D.J. , Smemo, K.A. , López‐Gutiérrez, J.C. & DeForest, J.L. (2012) Soil fungi influence the distribution of microbial functional groups that mediate forest greenhouse gas emissions. Soil Biology and Biochemistry, 53, 112–119.

[emi413152-bib-0003] Cannone, N. & Pignatti, S. (2014) Ecological responses of plant species and communities to climate warming: upward shift or range filling processes? Climatic Change, 123, 201–214.

[emi413152-bib-0004] Cannone, N. , Sgorbati, S. & Guglielmin, M. (2007) Unexpected impacts of climate change on alpine vegetation. Frontiers in Ecology and the Environment, 5, 360–364.

[emi413152-bib-0005] Cavaleri, M.A. , Reed, S.C. , Smith, W.K. & Wood, T.E. (2015) Urgent need for warming experiments in tropical forests. Global Change Biology, 21, 2111–2121. 25641092 10.1111/gcb.12860

[emi413152-bib-0006] Cavicchioli, R. , Thomas, T. & Curmi, P.M.G. (2000) Cold stress response in archaea. Extremophiles, 4, 321–331. 11139074 10.1007/s007920070001

[emi413152-bib-0007] Clark, I.M. , Hughes, D.J. , Fu, Q. , Abadie, M. & Hirsch, P.R. (2021) Metagenomic approaches reveal differences in genetic diversity and relative abundance of nitrifying bacteria and archaea in contrasting soils. Scientific Reports, 11, 1–9. 34354121 10.1038/s41598-021-95100-9 PMC8342464

[emi413152-bib-0008] D'Alò, F. , Baldrian, P. , Odriozola, I. , Morais, D. , Větrovský, T. , Zucconi, L. et al. (2022) Composition and functioning of the soil microbiome in the highest altitudes of the Italian Alps and potential effects of climate change. FEMS Microbiology Ecology, 98, fiac025. 35238906 10.1093/femsec/fiac025

[emi413152-bib-0009] D'Alò, F. , Odriozola, I. , Baldrian, P. , Zucconi, L. , Ripa, C. , Cannone, N. et al. (2021) Microbial activity in alpine soils under climate change. Science of the Total Environment, 783, 147012. 33872894 10.1016/j.scitotenv.2021.147012

[emi413152-bib-0010] DeLong, E.F. & Pace, N.R. (2001) Environmental diversity of bacteria and archaea. Systematic Biology, 50, 470–478. 12116647

[emi413152-bib-0011] Diaz, H.F. , Grosjean, M. & Graumlich, L. (2003) Climate variability and change in high elevation regions: past, present and future. Climatic Change, 59, 1–4.

[emi413152-bib-0012] Donhauser, J. & Frey, B. (2018) Alpine soil microbial ecology in a changing world. FEMS Microbiology Ecology, 94, fiy099. 10.1093/femsec/fiy099 30032189

[emi413152-bib-0046] Eddy, S.R. (2011) Accelerated profile HMM searches. PLoS Computational Biology, 7, e1002195 10.1371/journal.pcbi.1002195 PMC3197634 22039361

[emi413152-bib-0013] Grigoriev, I.V. , Nikitin, R. , Haridas, S. , Kuo, A. , Ohm, R. , Otillar, R. et al. (2014) MycoCosm portal: gearing up for 1000 fungal genomes. Nucleic Acids Research, 42, D699–D704. 24297253 10.1093/nar/gkt1183 PMC3965089

[emi413152-bib-0014] Hansman, R.L. , Griffin, S. , Watson, J.T. , Druffel, E.R. , Ingalls, A.E. , Pearson, A. et al. (2009) The radiocarbon signature of microorganisms in the mesopelagic ocean. Proceedings of the National Academy of Sciences, 106, 6513–6518. 10.1073/pnas.0810871106 PMC2672484 19366673

[emi413152-bib-0015] Hedderich, R. & Whitman, W.B. (2006) Physiology and biochemistry of the methane‐producing archaea. The Prokaryotes, 2, 1050–1079.

[emi413152-bib-0016] Hofmann, K. , Lamprecht, A. , Pauli, H. & Illmer, P. (2016) Distribution of prokaryotic abundance and microbial nutrient cycling across a high‐alpine altitudinal gradient in the Austrian Central Alps is affected by vegetation, temperature, and soil nutrients. Microbial Ecology, 72, 704–716. 27401822 10.1007/s00248-016-0803-z

[emi413152-bib-0017] IPCC . (2018) Summary for policymakers. In: Global warming of 1.5°C. Geneva: IPCC.

[emi413152-bib-0018] Isoda, R. , Hara, S. , Tahvanainen, T. & Hashidoko, Y. (2017) Comparison of archaeal communities in mineral soils at a boreal forest in Finland and a cold‐temperate forest in Japan. Microbes and Environments ME17100., 32, 390–393. 10.1264/jsme2.ME17100 PMC5745025 29109334

[emi413152-bib-0019] Kakuk, B. , Wirth, R. , Maróti, G. , Szuhaj, M. , Rakhely, G. , Laczi, K. et al. (2021) Early response of methanogenic archaea to H2 as evaluated by metagenomics and metatranscriptomics. Microbial Cell Factories, 20, 1–18. 34217274 10.1186/s12934-021-01618-y PMC8254922

[emi413152-bib-0020] Kanehisa, M. & Goto, S. (2000) KEGG: Kyoto encyclopedia of genes and genomes. Nucleic Acids Research, 28, 27–30. 10592173 10.1093/nar/28.1.27 PMC102409

[emi413152-bib-0021] Klein, J.A. , Harte, J. & Zhao, X.Q. (2005) Dynamic and complex microclimate responses to warming and grazing manipulations. Global Change Biology, 11, 1440–1451.

[emi413152-bib-0022] Korzhenkov, A.A. , Toshchakov, S.V. , Bargiela, R. , Gibbard, H. , Ferrer, M. , Teplyuk, A.V. et al. (2019) Archaea dominate the microbial community in an ecosystem with low‐to‐moderate temperature and extreme acidity. Microbiome, 7, 1–14. 30691532 10.1186/s40168-019-0623-8 PMC6350386

[emi413152-bib-0045] Könneke, M. , Bernhard, A.E. , de La Torre, J.R. , Walker, C.B. , Waterbury, J.B. , & Stahl, D.A. (2005) Isolation of an autotrophic ammonia‐oxidizing marine archaeon. Nature, 437, 543–546. 10.1038/nature03911 16177789

[emi413152-bib-0023] Langmead, B. & Salzberg, S.L. (2012) Fast gapped‐read alignment with bowtie 2. Nature Methods, 9, 357–359. 22388286 10.1038/nmeth.1923 PMC3322381

[emi413152-bib-0024] Leininger, S. , Urich, T. , Schloter, M. , Schwark, L. , Qi, J. , Nicol, G.W. et al. (2006) Archaea predominate among ammonia‐oxidizing prokaryotes in soils. Nature, 442, 806–809. 16915287 10.1038/nature04983

[emi413152-bib-0025] Li, D. , Liu, C.M. , Luo, R. , Sadakane, K. & Lam, T.W. (2015) MEGAHIT: an ultra‐fast single‐node solution for large and complex metagenomics assembly via succinct de Bruijn graph. Bioinformatics, 31, 1674–1676. 25609793 10.1093/bioinformatics/btv033

[emi413152-bib-0026] Lyu, Z. , Chou, C.W. , Shi, H. , Wang, L. , Ghebreab, R. , Phillips, D. et al. (2018) Assembly of methyl coenzyme M reductase in the methanogenic archaeon Methanococcus maripaludis. Journal of Bacteriology, 200, e00746–e00717. 29339414 10.1128/JB.00746-17 PMC5847650

[emi413152-bib-0027] Malfasi, F. & Cannone, N. (2021) Phytosociology of the vegetation communities of the Stelvio pass area. Journal of Maps, 17, 367–375.

[emi413152-bib-0028] Metcalf, W.W. , Griffin, B.M. , Cicchillo, R.M. , Gao, J. , Janga, S.C. , Cooke, H.A. et al. (2012) Synthesis of methylphosphonic acid by marine microbes: a source for methane in the aerobic ocean. Science, 337(6098), 1104–1107. 22936780 10.1126/science.1219875 PMC3466329

[emi413152-bib-0029] Meyer, F. , Paarmann, D. , D'Souza, M. , Olson, R. , Glass, E.M. , Kubal, M. et al. (2008) The metagenomics RAST server–a public resource for the automatic phylogenetic and functional analysis of metagenomes. BMC Bioinformatics, 9, 1–8. 18803844 10.1186/1471-2105-9-386 PMC2563014

[emi413152-bib-0030] Neilson, J.W. , Califf, K. , Cardona, C. , Copeland, A. , van Treuren, W. , Josephson, K.L. et al. (2017) Significant impacts of increasing aridity on the arid soil microbiome. mSystems, 2, e00195–e00116. 28593197 10.1128/mSystems.00195-16 PMC5451488

[emi413152-bib-0031] Prestat, E. , David, M.M. , Hultman, J. , Taş, N. , Lamendella, R. , Dvornik, J. et al. (2014) FOAM (functional ontology assignments for metagenomes): a hidden Markov model (HMM) database with environmental focus. Nucleic Acids Research, 42, e145. 25260589 10.1093/nar/gku702 PMC4231724

[emi413152-bib-0048] R Core Team. (2020) R: A language and environment for statistical computing. Vienna: R Foundation for Statistical Computing. https://www.R-project.org/. R Foundation for Statistical Computing

[emi413152-bib-0032] Sannino, C. , Cannone, N. , D'Alò, F. , Franzetti, A. , Gandolfi, I. , Pittino, F. et al. (2022) Fungal communities in European alpine soils are not affected by short‐term in situ simulated warming than bacterial communities. Environmental Microbiology, 24, 4178–4192. 35691701 10.1111/1462-2920.16090

[emi413152-bib-0033] Souza, R.C. , Mendes, I.C. , Reis‐Junior, F.B. , Carvalho, F.M. , Nogueira, M.A. , Vasconcelos, A.T.R. et al. (2016) Shifts in taxonomic and functional microbial diversity with agriculture: how fragile is the Brazilian Cerrado? BMC Microbiology, 16, 1–15. 26983403 10.1186/s12866-016-0657-z PMC4794851

[emi413152-bib-0034] Stahl, D.A. & de la Torre, J.R. (2012) Physiology and diversity of ammonia‐oxidizing archaea. Annual Review of Microbiology, 66, 83–101. 10.1146/annurev-micro-092611-150128 22994489

[emi413152-bib-0035] Starke, R. , Siles, J.A. , Fernandes, M.L.P. , Schallert, K. , Benndorf, D. , Plaza, C. et al. (2021) The structure and function of soil archaea across biomes. Journal of Proteomics, 237, 104147. 33582288 10.1016/j.jprot.2021.104147

[emi413152-bib-0036] Thompson, L.G. (2000) Ice core evidence for climate change in the tropics: implications for our future. Quaternary Science Reviews, 19, 19–35.

[emi413152-bib-0037] Timonen, S. & Bomberg, M. (2009) Archaea in dry soil environments. Phytochemistry Reviews, 8, 505–518.

[emi413152-bib-0038] Treusch, A.H. , Leininger, S. , Kletzin, A. , Schuster, S.C. , Klenk, H.P. & Schleper, C. (2005) Novel genes for nitrite reductase and Amo‐related proteins indicate a role of uncultivated mesophilic crenarchaeota in nitrogen cycling. Environmental Microbiology, 7, 1985–1995. 16309395 10.1111/j.1462-2920.2005.00906.x

[emi413152-bib-0039] Venter, J.C. , Remington, K. , Heidelberg, J.F. , Halpern, A.L. , Rusch, D. , Eisen, J.A. et al. (2004) Environmental genome shotgun sequencing of the Sargasso Sea. Science, 304, 66–74. 15001713 10.1126/science.1093857

[emi413152-bib-0040] Yu, Y. , Liu, L. , Wang, J. , Zhang, Y. & Xiao, C. (2021) Effects of warming on the bacterial community and its function in a temperate steppe. Science of the Total Environment, 792, 148409. 34146803 10.1016/j.scitotenv.2021.148409

[emi413152-bib-0041] Yuan, M.M. , Guo, X. , Wu, L. , Zhang, Y. , Xiao, N. , Ning, D. et al. (2021) Climate warming enhances microbial network complexity and stability. Nature Climate Change, 11, 343–348.

[emi413152-bib-0042] Žifčáková, L. , Větrovský, T. , Lombard, V. , Henrissat, B. , Howe, A. & Baldrian, P. (2017) Feed in summer, rest in winter: microbial carbon utilization in forest topsoil. Microbiome, 5, 1–12. 28923122 10.1186/s40168-017-0340-0 PMC5604414

