# Supplementary material for: Effects of 5‐year experimental warming in the Alpine belt on soil Archaea: Multi‐omics approaches and prospects
Source: Environ Microbiol Rep. 2023 Mar 30;15(4):291–7. doi: 10.1111/1758-2229.13152 (PMC10316362; doi:10.1111/1758-2229.13152)
Supplement: Supplementary file 1 — Data S1: Supporting Information [file EMI4-15-291-s001.docx]

**Supplementary information**

**Title:**

**Effects of five-year experimental warming in the Alpine belt on soil Archaea: multi-omics approaches and prospects**

Running title: Archaeal response to warming in the Alpine belt

Federica D’Alò^1^, Laura Zucconi^1,2,*^, Silvano Onofri^1^, Fabiana Canini^1^, Nicoletta Cannone^3^, Francesco Malfasi^3^, Daniel Kumazawa Morais^4,5,^ Robert Starke^6^

^1^ Department of Ecological and Biological Sciences, University of Tuscia, Largo dell’Università, 01100 Viterbo, Italy

^2^ Institute of Polar Sciences, National Research Council of Italy (CNR-ISP), Spianata S. Raineri 86, 98122 Messina, Italy

^3^ Department of Science and High Technology, Insubria University, Via Valleggio, 11, 21100 Como (CO), Italy.

^4^Biological Institute of São Paulo – Vila Mariana, Av. Conselheiro Rodrigues Alves 1252, São Paulo, SP 04014-002, Brazil

^5^Norwegian College of Fishery Science, UiT the Arctic University of Norway, Muninbakken 21, Tromsø 9019, Norway

^6^ Institute of Microbiology of the Czech Academy of Sciences, Vídeňská 1083, 14220 Praha 4, Czech Republic

Keywords: metagenomics, metatranscriptomics, experimental warming, archaea, soil microbiome

The authors declare no conflict of interest.

# Materials & Methods

*Sampling sites and design*

The metagenomic and metatranscriptomic raw data were obtained from a previously published paper focusing on the abundance, diversity, and function of bacteria and fungi (D’Alò *et al.*, 2022). Briefly, the selected area was located around the Stelvio Pass in the Stelvio National Park in the central Italian Alps (46°31’ N, 10°25’ E) due to evidence of recent climate change, which included vegetational changes since 1953, such as shrub upwards migration and encroachment at the expense of Alpine grasslands and snowbeds (Cannone *et al.*, 2007). The selected Alpine belt (2604-2624 m a.s.l.) comprised two different plant communities: a climax Alpine grassland (*Caricetum curvulae*) and an Alpine snowbed (*Salicetum herbaceae*). Both plant communities are highly vulnerable to the impacts of climate change and are at high risk of regression as a result of the ingression of species from neighboring communities (Cannone *et al.*, 2007; Cannone and Pignatti, 2014; Malfasi and Cannone, 2021). To study the effect of short-term warming, a manipulative warming experiment was conducted (D’Alò *et al.*, 2021). In 2014, small hexagonal OTCs (2.08 m in diameter) were installed in both Alpine grassland and Alpine snowbed. OTCs passively increased the ambient summer temperature to +1 °C (Malfasi and Cannone, 2021) to reach values predicted by the best future warming scenario (RCP2.6) by 2100 (IPCC, 2018). The paired plots outside the OTCs were used as controls, representing current conditions. Five soil samples were collected for each condition on the same day in July 2019 during the peak vegetation season. The same samples used in this study have already been used for enzyme activity, qPCR and total microbial community analysis (D’Alò *et al.*, 2021, 2022). For each sample, soil was collected with a sterilized spatula from at least three points for each condition down to a depth of 10 cm. After removal of plant debris and roots, the soil was passed through a 5-mm sterile mesh and mixed to obtain a composite sample, which was collected in Falcon tubes. Each Falcon tube was frozen in liquid nitrogen, transported to the laboratory under frozen conditions, and stored at -80°C until analysis.

*Soil physicochemical parameters*

Soil temperatures were recorded in situ at 2cm depth using thermistors (Hobo pro V2 2x U23-003, Onset Corp., Ma, USA). Soil pH of the soil was determined by adding 20mL of distilled water to 10g of air-dried soil and vigorously shaking the mixture. Soils oven-dried at 105°C were used to calculate water content. All chemical analyses were carried out on the <2mm soil fraction. An automatic elemental analyzer was used to determine the total C, N, and H contents (Carlo Erba). To determine dissolved nutrient concentrations, 10 g of airdried soil was mixed in 100 ml deionized water, shaken for 2 h, and filtered using glass microfiber filters (Whatman GF/D). The obtained extracts were analyzed colorimetrically for ammonium using the salicylate method, nitrate using the cadmium reduction method, and phosphorus using the molybdenum blue method and a continuous flow analyzer (FlowSys, Systea, Rome, Italy). Ca, K and Mg concentration was determined by (K) concentrations were determined by atomic absorption spectrophotometry after digestion with H_2_SO_4_. Soil electrical conductivity determination was carried from solutions made by mixing 10 g soil sample with 25 ml distilled water shaking for 3 min and laying them aside for 30 min.

*Nucleic acid extraction and library preparation*

Three 1g aliquots per sample were homogenized using mortar and pestle under liquid nitrogen. DNA and RNA were co-extracted using the DNA Elution Accessory Kit combined with the RNeasy PowerSoil Total RNA Kit (MolBio Laboratories). Total DNA was cleaned by a GeneClean Turbo Kit (MP Biomedicals), checked for quality and length distribution on an Agilent 2100 Bioanalyzer (Agilent Technologies), and used to generate the metagenomic libraries using the TruSeq Nano DNA Library Preparation Kit. The library size distribution was re-checked using an Agilent 2100 Bioanalyzer (Agilent Technologies). Total RNA was purified using the OneStep PCR Inhibitor Removal kit (ZymoResearch), and DNA was removed using a DNA-free DNA Removal kit (Thermo Fisher Scientific) and checked by negative PCR results using the bacterial primers 515F and 806R (Caporaso *et al.*, 2012). This product was checked for quality (RNA integrity number) and length distribution on an Agilent 2100 Bioanalyzer (Agilent Technologies). Approximately 1 μg of RNA was treated with an equimolar mixture of RiboZero rRNA Removal Kits Human‐Mouse‐Rat and Bacteria (Epicenter) to remove both prokaryotic and eukaryotic rRNAs. rRNA removal was checked on an Agilent 2100 Bioanalyzer (Žifčáková *et al.*, 2016). Reverse transcription was performed using SuperScript III (Thermo Fisher Scientific). The TruSeq Stranded Total RNA kit was used to generate metatranscriptomic libraries with final 14 cycles of amplification by FailSafe PCR Enzyme (Lucigen), and the library size distribution was rechecked on an Agilent 2100 Bioanalyzer (Agilent Technologies).

*Quantification of bacterial and fungal gene copy numbers*

For all samples, quantitative PCR (Applied Biosystems StepOnePlus cycler) on bacterial and fungal rDNA copies was performed as described before (D’Alò *et al.*, 2021). The primers 1108f and 1132r were used for bacteria (Wilmotte *et al.*, 1993; Amann *et al.*, 1995) and FR1/FF390 for fungi (Chemidlin Prévost-Bouré *et al.*, 2011; Žifčáková *et al.*, 2016). Each 20 µl of reaction mixture contained 10 µl of SYBR Green Master Mix (Applied Biosystems), 0.9 µl of bovine serum albumin (BSA; 10 mg/ml), 1.35 µl of each primer, 1.5 µl of template, and 6.1 µl of water. The qPCR cycling protocol for DNA quantification was as follows: 56 °C for 2 min, 95 °C for 10 min, 95 °C for 15 s, and 60 °C for 1 min (40 cycles). Genomic DNA from *Streptomyces lincolnensis* DNS 40335 served as bacterial standard and from *Hypholoma fasciculare* CCBAS281 as fungal one. Bacterial and fungal abundances were expressed as the number of copies of rDNA genes per gram of dry soil.

*Sequencing and bioinformatic analysis*

Samples of the metagenome (MG) and metatranscriptome (MT) were pooled in equimolar volumes and sequenced on an Illumina NovaSeq6000 SP (DS-150) with a 2 x 150 bp at an external laboratory (SEQme, Czech Republic). MG assembly and annotation were performed as previously described (Žifčáková *et al.*, 2017). Briefly, Trimmomatic 0.36 (Bolger *et al.*, 2014) and FASTX-Toolkit (http://hannonlab.cshl.edu/fastx_toolkit/) were used to remove adaptor contamination, trim low-quality ends of reads, and omit reads with overall low quality (<30); sequences shorter than 50 bp were omitted. mRNA reads were filtered from the files using the bbduk.sh (version 38.26) program in BBTools (https://sourceforge.net/projects/bbmap/). Combined assembly was performed using MEGAHIT 1.1.3 (Li *et al.*, 2015). Gene calling was performed using MG-RAST (Meyer *et al.,* 2008) where all the assemblies of MG and MT were deposited (mgm4901504.3 and mgm4901505.3, respectively). Taxonomic identification was performed both using MG-RAST identification based on IMG (Integrated Microbial Genomes) dataset as a reference and by BLAST against all published fungal genomes available in MycoCosm fungal genomics portal on January 2020 (Grigoriev *et al.*, 2014). Of these two, that taxonomic identification with a higher bitscore was used as the best hit with minimal threshold of bitscore 54. The functions of the predicted genes were annotated with the hmmsearch function in HMMER 3.2.1 (Eddy, 2011) using the FOAM database as a source of HMMs for relevant genes (Prestat *et al.*, 2014). To obtain the number of genes per sample, reads were mapped to the assembled contigs using the Bowtie 2 program (Langmead and Salzberg, 2012). Using the Kyoto Encyclopedia of Genes and Genomes (KEGG) Orthology database (Kanehisa and Goto, 2000), the dominant pathways were annotated at KEGG level 2. All analyses were performed in R (R Development Core Team, 2019). Visualizations were performed with ggplot2 (Wickham, 2017) using the function *stat_compare_means* for paired t-tests and the function *corr* for correlation analysis. Different methods to estimate correlation yielded similar results which is why we used Spearman (**Supplementary Figure 1**).

# References

Amann, R., Ludwig, W., and Schleifer, K. (1995) Phylogenetic identification and in situ detection of individual microbial cells without cultivation. Microbiological reviews, 59: 143-169

Bolger, A.M., Lohse, M., and Usadel, B. (2014) Trimmomatic: A flexible trimmer for Illumina sequence data. Bioinformatics 30: 2114-2120.

Cannone, N., and Pignatti, S. (2014) Ecological responses of plant species and communities to climate warming: Upward shift or range filling processes? Climatic Change 123: 201-214.

Cannone, N., Sgorbati, S., and Guglielmin, M. (2007) Unexpected impacts of climate change on alpine vegetation. Frontiers in Ecology and the Environment 5: 360-364.

Caporaso, J.G., Lauber, C.L., Walters, W.A., Berg-Lyons, D., Huntley, J., Fierer, N., et al. (2012) Ultra-high-throughput microbial community analysis on the Illumina HiSeq and MiSeq platforms. ISME Journal 6: 1621-1624.

Chemidlin Prévost-Bouré, N., Christen, R., Dequiedt, S., Mougel, C., Lelièvre, M., Jolivet, C., et al. (2011) Validation and application of a PCR primer set to quantify fungal communities in the soil environment by real-time quantitative PCR. PLoS ONE **6**.

D’Alò, F., Baldrian, P., Odriozola, I., Morais, D., Větrovský, T., Zucconi, L., et al. (2022) Composition and functioning of the soil microbiome in the highest altitudes of the Italian Alps and potential effects of climate change. FEMS Microbiology Ecology 98, fiac025.

D’Alò, F., Odriozola, I., Baldrian, P., Zucconi, L., Ripa, C., Cannone, N., et al. (2021) Microbial activity in alpine soils under climate change. Science of the Total Environment 783, 147012.

Eddy, S.R. (2011) Accelerated profile HMM searches. PLoS Computational Biology 7: e1002195.

Grigoriev, I. v., Nikitin, R., Haridas, S., Kuo, A., Ohm, R., Otillar, R., et al. (2014) MycoCosm portal: Gearing up for 1000 fungal genomes. Nucleic Acids Research 42: D699-D704.

IPCC (2018) Summary for Policymakers. In: Global Warming of 1.5°C.

Kanehisa, M. and Goto, S. (2000) KEGG: Kyoto Encyclopedia of Genes and Genomes. Nucleic Acids Research 28: 27–30.

Langmead, B., and Salzberg, S.L. (2012) Fast gapped-read alignment with Bowtie 2. Nature Methods 9:357.

Li, D., Liu, C.M., Luo, R., Sadakane, K., and Lam, T.W. (2015) MEGAHIT: An ultra-fast single-node solution for large and complex metagenomics assembly via succinct de Bruijn graph. Bioinformatics 31: 1674-1676.

Malfasi, F. and Cannone, N. (2021) Phytosociology of the vegetation communities of the Stelvio Pass area. Journal of Maps 17: 367-375.

Meyer, F., Paarmann, D., D'Souza, M., Olson, R., Glass, E.M., Kubal, M., et al. (2008). The metagenomics RAST server–a public resource for the automatic phylogenetic and functional analysis of metagenomes. BMC bioinformatics, 9: 1-8.

Prestat, E., David, M.M., Hultman, J., Taş, N., Lamendella, R., Dvornik, J., et al. (2014) FOAM (Functional Ontology Assignments for Metagenomes): A Hidden Markov Model (HMM) database with environmental focus. Nucleic Acids Research 42: e145.

R Development Core Team (2019) R Core Team (2020). R: A language and environment for statistical computing. R Foundation for Statistical Computing, Vienna, Austria. URL https://www.R-project.org/. R Foundation for Statistical Computing.

Wickham, H. (2017) ggplot2: Elegant Graphics for Data Analysis. Journal of Statistical Software.

Wilmotte, A., van der Auwera, G., and de Wachter, R. (1993) Structure of the 16 S ribosomal RNA of the thermophilic cyanobacterium chlorogloeopsis HTF ('mastigocladus laminosus HTF’) strain PCC7518, and phylogenetic analysis. FEBS Letters 317: 96-100.

Žifčáková, L., Větrovský, T., Howe, A., and Baldrian, P. (2016) Microbial activity in forest soil reflects the changes in ecosystem properties between summer and winter. Environmental Microbiology 18: 288-301.

Žifčáková, L., Větrovský, T., Lombard, V., Henrissat, B., Howe, A., and Baldrian, P. (2017) Feed in summer, rest in winter: microbial carbon utilization in forest topsoil. Microbiome 5: 1-12.

# Supplementary figures & tables


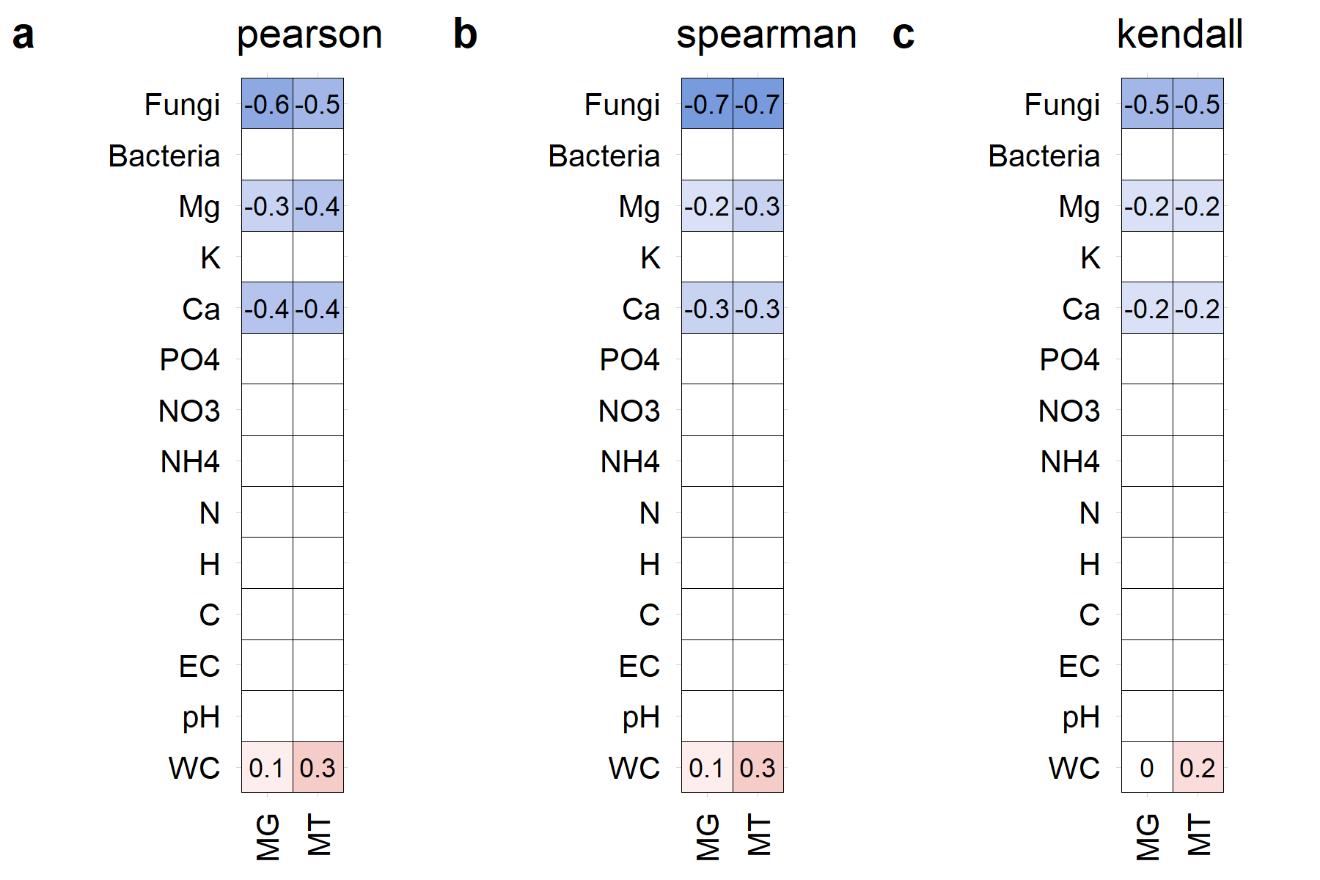


**Supplementary Figure 1:** Different methods to estimate correlation between soil physicochemical parameters and abundance of archaeal genes and transcript.


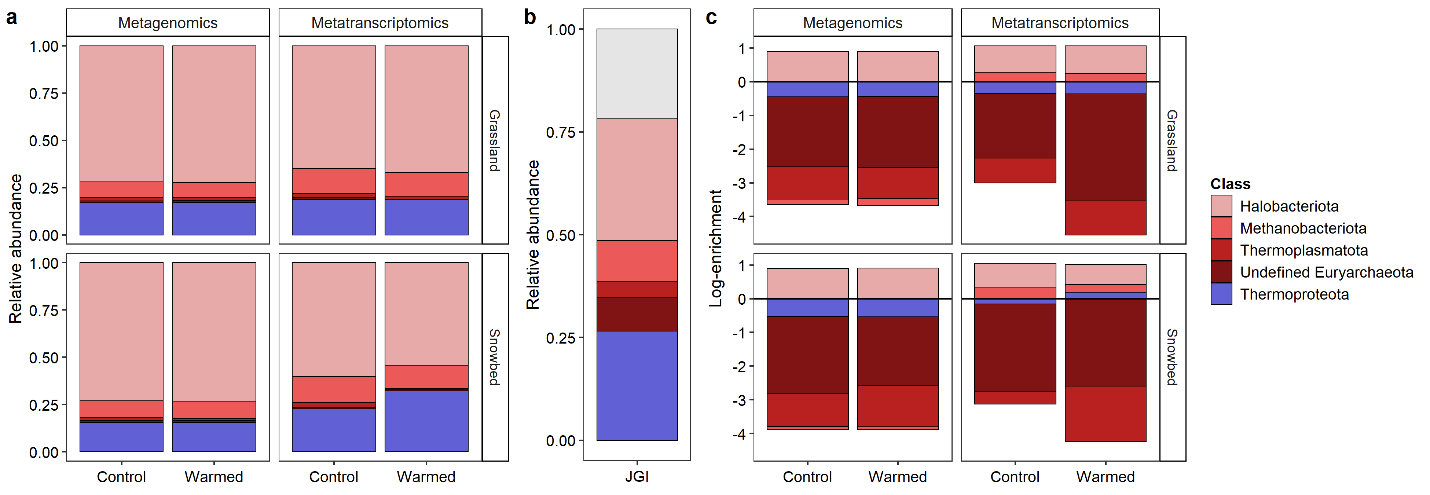


**Supplementary Figure 2:** Relative abundance of archaeal classes in all genes and transcripts in grasslands and snowbeds in control plots and during warming (a), in the JGI database (b) and their enrichment in the data with respect to the database (c).

**Supplementary Table 1:** Average (AV) and standard deviation (SD) of edaphic and environmental variables with P-value (p) and effect size (effsize) from the Kruskal-Wallis test for the four-way comparison. The unit of WC is %, EC is µS, C is %, H is %, N is %, NH_4_ is ppm, NO_3_ is ppm, PO_4_ is ppm, Ca is ppm, K is ppm, Mg is ppm, bacteria and fungi are rDNA copy numbers.

| Veg | Grassland | | | | Snowbed | | | | Kruskal-Wallis | |
| --- | --- | --- | --- | --- | --- | --- | --- | --- | --- | --- |
| Treat | Control | | Warmed | | Control | | Warmed | |  |  |
|  | AV | SD | AV | SD | AV | SD | AV | SD | p | effsize |
| WC | 38,65 | 5,18 | 39,50 | 5,75 | 41,74 | 4,60 | 43,33 | 1,02 | 0,520 | -0,046 |
| pH | 5,12 | 0,28 | 5,26 | 0,37 | 5,34 | 0,53 | 5,44 | 0,26 | 0,520 | -0,046 |
| EC | 99,54 | 36,18 | 114,10 | 45,39 | 88,92 | 39,10 | 106,64 | 32,33 | 0,193 | 0,108 |
| C | 7,32 | 1,27 | 7,66 | 2,70 | 8,16 | 1,75 | 8,80 | 0,52 | 0,408 | -0,006 |
| H | 1,38 | 0,22 | 1,41 | 0,41 | 1,55 | 0,25 | 1,65 | 0,07 | 0,452 | -0,023 |
| N | 0,50 | 0,06 | 0,51 | 0,11 | 0,41 | 0,11 | 0,54 | 0,06 | 0,168 | 0,128 |
| NH_4_ | 5,85 | 2,20 | 6,28 | 2,15 | 3,38 | 0,78 | 5,13 | 1,92 | 0,113 | 0,186 |
| NO_3_ | 1,18 | 0,90 | 1,24 | 1,57 | 1,39 | 0,48 | 1,55 | 0,85 | 0,488 | -0,036 |
| PO_4_ | 5,27 | 3,52 | 5,74 | 3,09 | 1,87 | 1,38 | 1,03 | 0,41 | 0,005 | 0,629 |
| Ca | 90,05 | 60,18 | 97,63 | 37,06 | 59,43 | 28,21 | 63,63 | 22,09 | 0,374 | 0,007 |
| K | 18,87 | 4,96 | 29,57 | 14,28 | 7,46 | 2,25 | 10,25 | 2,39 | 0,002 | 0,746 |
| Mg | 26,51 | 15,82 | 32,89 | 15,38 | 15,23 | 7,40 | 19,67 | 8,46 | 0,141 | 0,154 |
| Bacteria | 4,51E+12 | 1,36E+12 | 3,89E+12 | 6,63E+11 | 5,64E+12 | 8,35E+11 | 5,97E+12 | 8,81E+11 | 0,022 | 0,413 |
| Fungi | 1,22E+10 | 5,27E+09 | 1,33E+10 | 7,98E+09 | 8,35E+09 | 2,47E+09 | 6,12E+09 | 1,95E+09 | 0,114 | 0,185 |
